# An Adaptive Grouping Method for Efficient Multi-Beam Testing of Phased Arrays

**DOI:** 10.3390/s26175391

**Published:** 2026-08-26

**Authors:** Chen Yan, Junhao Zheng, Huaqiang Gao, Xiaoming Chen

**Affiliations:** School of Information and Communications Engineering, Xi’an Jiaotong University, Xi’an 710049, China; chen.yan@stu.xjtu.edu.cn (C.Y.); 1071218679@stu.xjtu.edu.cn (J.Z.); huaqiang.gao@xjtu.edu.cn (H.G.)

**Keywords:** antenna measurement, beam steering, *K*-means clustering, phased array antenna, radiation pattern

## Abstract

Phased arrays are key for next-generation mobile communication, satellite communication, and radar systems. Radiation patterns are essential performance metrics for evaluating antennas, but conventional mechanically scanned measurements become inefficient when repeated for every beam of the phased array. Existing active-element-pattern-based methods reduce this burden by grouping elements with similar coupling environments, yet their grouping and representative-element selection are mainly based on physical intuition, and on–off-mode-based measurements may differ from the actual all-on operating state. This paper proposes a data-driven adaptive grouping method based on *K*-means unsupervised learning. Element positions and broadside phase information construct the feature matrix, and a composite evaluation function considering group size and electric-field variation subdivides high-dynamic regions. Representative element patterns are acquired in the all-on mode, with sparse angular sampling to further reduce measurement time. Simulation results of an 8×8 dipole array show that the proposed method reconstructs multi-beam array patterns using 16 representative elements, fewer than the 25 representative elements required by a 5×5 grouping strategy, with the same accuracy in the main lobe and improved accuracy in sidelobe nulls. The effectiveness of the proposed grouping method has also been demonstrated for the measured 4×4 mmWave phased array.

## 1. Introduction

Phased array antennas have become one of the key enablers in modern wireless systems, with applications ranging from fifth-generation (5G) and future sixth-generation (6G) terrestrial networks to satellite sensing/communication platforms [1,2,3,4]. In millimeter-wave (mmWave) 5G systems, phased arrays with beamforming capability concentrate radiated energy toward target users and are therefore essential for compensating the severe free-space path loss inherent to mmWave frequencies. In satellite communications, phased arrays with multi-beam scanning capability also play an important role in high-throughput satellites and low-Earth-orbit satellite systems. The wide deployment of phased arrays in these diverse and demanding scenarios makes accurate characterization of their radiation performance increasingly important.

Conventional fixed-beam radiated measurements are commonly performed using near-field [5,6], direct far-field [7], compact antenna test range (CATR) [8], plane-wave generator (PWG) [9], or other similar indirect far-field techniques. Unlike fixed-beam antennas, however, phased arrays can scan beams over many directions (i.e., steering states), and each steering state requires a radiation-pattern measurement. For large-scale phased arrays, the number of required beam states can easily reach hundreds or even thousands [10]. If a conventional single-pattern measurement procedure is mechanically repeated over the required angular range for each beam independently, the overall measurement process becomes extremely time-consuming and inefficient. Therefore, efficient multi-beam pattern measurement methods that can substantially reduce total measurement time while maintaining high accuracy are of practical significance for multi-beam testing of phased arrays.

A number of studies have investigated multi-beam measurement for phased arrays [10,11,12,13,14,15,16,17]. A typical class of methods measures the radiation pattern of each antenna element and then reconstructs the array pattern for arbitrary beam directions [10,11,12]. For example, a compressed-sensing method based on two-step iterative shrinkage [6] was introduced in [11] to improve sparse pattern reconstruction and enhance sidelobe accuracy. The measured element patterns in [10,11,12] implicitly refer to the active element pattern (AEP) under practical mutual coupling [13]. To further speed up multi-beam tests, array elements can be classified into groups according to their coupling environments, assuming that elements with similar coupling conditions have similar element patterns [14,15,16,17]. For instance, interior elements, edge elements, and corner elements are often treated as separate categories because their local electromagnetic environments are physically different.

Although these approaches reduce measurement effort, several limitations remain. In most existing grouping strategies, the grouping of array elements and the selection of representative elements rely mainly on physical intuition about coupling environments, rather than on data-driven analysis of the actual electromagnetic field distribution. The representative elements are often selected manually. Moreover, some grouping methods treat the corner elements of a planar array as independent groups. For an 8×8 planar array, for example, the four corner elements may be assigned to four different groups. However, because these corner elements have similar coupling environments and related pattern symmetry, it is feasible to select one representative corner element and recover the remaining corner-element patterns through rotation, symmetry mapping, and phase compensation. This treatment can reduce the number of elements that need to be measured. In addition, the element patterns in [17] are obtained in the on–off mode, where only the element under test is activated. In contrast, the comparison reported in [18] shows that, for a 4×4 mmWave phased array, the all-on mode, in which all elements are simultaneously excited, gives smaller measurement errors: the amplitude and phase errors are approximately 0.1 dB and $1^{\circ }$, respectively, compared with 0.8 dB and $9^{\circ }$ in the on–off mode. These results indicate that all-on element-pattern acquisition is necessary for accurate mmWave phased array pattern reconstruction.

Based on the above analysis, this paper proposes an adaptive grouping method using *K*-means-based unsupervised clustering [19,20,21]. The position information of each array element and the phase information extracted from the broadside array pattern are used to construct the feature matrix. A composite evaluation function, which considers both the number of elements in each group and the degree of electric-field variation, is then used to identify and further subdivide groups located in high-dynamic field regions. This procedure enables effective element classification and automatic representative-element selection. In the proposed method, corner elements with similar electromagnetic environments can be assigned to the same group, reducing the number of representative elements that must be measured. For example, for an 8×8 dipole array, the proposed algorithm obtains 16 groups, fewer than the 25 groups used in the 5×5 grouping strategy of [17], while achieving comparable overall accuracy and improving the null-depth accuracy of some minor lobes, which is an important consideration in satellite communication systems. Unlike [11], which sparsely samples all element patterns to reduce the number of samples per element without reducing measured elements, this work improves efficiency by measuring fewer elements, and the grouping method can be combined with sparse sampling for further time reduction. Furthermore, the representative element patterns are measured using the all-on method adopted in [11], and sparse angular sampling is introduced during pattern measurement to further reduce the measurement time. Both simulation and measurement results verify the effectiveness of the proposed method.

To make the contribution explicit, the novelty of this work is not simply the use of K-means clustering, but the construction of a measurement-oriented adaptive grouping workflow: (i) the broadside phase is corrected before feature extraction, (ii) high-dynamic groups are automatically subdivided using a size- and field-variation-based criterion, and (iii) representative active element patterns are acquired in the all-on mode and may be sparsely sampled to reduce test time.

## 2. Methods

This section presents the theoretical framework of the proposed adaptive grouping method. The method consists of three main parts: feature matrix construction, adaptive group subdivision based on a composite evaluation criterion, and array-pattern calculation from representative active element patterns. At the end of this section, the all-on measurement procedure used to acquire the representative element patterns is also introduced.

### 2.1. Feature Matrix Construction

The proposed method first extracts the co-polarized and cross-polarized electric field components at the element positions from the broadside far-field pattern of the array, which are denoted as $E_{n}^{co}$ and $E_{n}^{cross}$ for the *n*th element (*n* = 1, 2, …, *N*), respectively. The position of the *n*th element is denoted by $r_{n}=\left(x_{n},y_{n}\right)$, where $k_{0}$ represents the wavenumber in free space. According to a previous study [22], the phase of the antenna element pattern varies too rapidly when the common phase factor is retained, which makes direct similarity extraction unreliable. Consequently, the spatial phase shift relative to the array center is removed prior to feature construction, as formulated in Equation (1). Under uniform amplitude excitation, the amplitude variation across elements is so small that it provides almost no discriminative information. In addition, removing the common propagation factor does not alter the field magnitudes or lose any information. (It is equivalent to remove the carrier frequency of a radio frequency signal to facilitate baseband processing). Only the co-polarized phase of the *n*th element is adopted as the feature for the subsequent initial clustering.

(1) $${\tilde{E}}_{n}^{co}=E_{n}^{co}\cdot e^{+jk_{0}\left|r_{n}\right|},\;{\tilde{E}}_{n}^{cross}=E_{n}^{cross}\cdot e^{+jk_{0}\left|r_{n}\right|}$$

For the *n*th element, the physical position and the corrected phase are used as the features for subsequent *K*-means clustering. For the *n*th element, the spatial and electrical features are formulated in Equations (2) and (3). Notably, the phase features of all elements can be extracted from a single additional complex far-field measurement of the complete array excited with equal amplitude and phase, without requiring *N* individual element measurements beforehand. The first feature indicates the normalized radial distance to the array center, while the second and third features correspond to the normalized distances to the array boundaries along the *x*- and *y*-axes, respectively. Additionally, the fourth feature denotes the phase of the co-polarized component with the common phase factor removed.

(2) $$f_{n}^{\left(1\right)}=\frac{\left|r_{n}\right|}{\max(\left|r_{n}\right|)},\;f_{n}^{\left(2\right)}=\frac{x_{\max}-\left|x_{n}\right|}{x_{\max}},\;f_{n}^{\left(3\right)}=\frac{y_{\max}-\left|y_{n}\right|}{y_{\max}}$$

(3) $$f_{n}^{\left(4\right)}=∠{\tilde{E}}_{n}^{co}$$

In Equation (2), $\max(\left|r_{n}\right|)$ is the maximum distance from the array center to any element, and $x_{\max}$ and $y_{\max}$ are the maximum element distances along the *x*- and *y*-axes. To avoid scale imbalance among different physical quantities, all four features are normalized before clustering.

The normalized feature vector of the *n*th element and the complete feature matrix of the array are then written as

(4) $$f_{n}={\left[{\hat{f}}_{n}^{\left(1\right)},{\hat{f}}_{n}^{\left(2\right)},{\hat{f}}_{n}^{\left(3\right)},{\hat{f}}_{n}^{\left(4\right)}\right]}^{T},F={\left[f_{1},f_{2},\ldots ,f_{N}\right]}^{T}$$

The matrix *F* contains both the spatial coordinates of the array elements and their corresponding phase information derived from the broadside far-field pattern. It is therefore used as the input feature matrix for the *K*-means clustering.

### 2.2. Adaptive Group Subdivision

The proposed method employs the *K*-means unsupervised learning algorithm to classify the array elements. An initial cluster number *k* is first selected; in this work, *k* = 4 is used as the initial number of groups. The initial clustering result is then refined by an adaptive subdivision process. This refinement is necessary because a group containing many elements, or a group located in a region with strong field variation, may still include elements with noticeably different active element patterns.

The first evaluation parameter $S_{k}$ is the relative size of each group. A large value indicates that the group contains a large fraction of the array elements and may require further subdivision.

(5) $$S_{k}=\frac{N_{k}}{\sum_{j=1}^{C_{k}}N_{j}}$$

In Equation (5), $N_{k}$ is the number of elements in the *k*th group, and $C_{k}$ is the current number of groups. In addition to the group size, the proposed method evaluates the field variation between adjacent groups. For the *k*th group, the representative element is selected as the element whose four-dimensional feature vector is closest to the cluster center of that group. The electric field gradient magnitude between two adjacent representative elements from groups $k_{1}$ and $k_{2}$ is defined as shown in Equation (6), where $E_{rep_{k}}^{co}$ and $E_{rep_{k}}^{cross}$ denote the co-polarized and cross-polarized electric field components of the *k*th representative element, respectively, which are extracted from the broadside far-field pattern of the array.

(6) $$g(k_{1},k_{2})=\sqrt{{\left|E_{rep_{k_{1}}}^{co}-E_{rep_{k_{2}}}^{co}\right|}^{2}+{\left|E_{rep_{k_{1}}}^{cross}-E_{rep_{k_{2}}}^{cross}\right|}^{2}}$$

The cumulative gradient around the representative element of the *k*th group $B_{k}$ is obtained by summing the absolute gradient values between this representative element and the representative elements of all adjacent groups, as shown in Equation (7).

(7) $$B_{k}=\sum_{(k,j)\in C_{k}}^{C_{k}}g(k,j)$$

Subsequently, the normalized gradient parameter $G_{k}$ for the *k*th group is then defined according to Equation (8).

(8) $$G_{k}=\frac{B_{k}}{\sum_{j=1}^{C_{k}}B_{j}}$$

A larger $G_{k}$ means that the electric field around the group varies more strongly and that the group is located in a high-dynamic region. Conversely, a smaller $G_{k}$ indicates that the group lies in a slowly varying region. By combining the group-size parameter and the normalized gradient parameter, the composite evaluation index for the *k*th group $J_{k}$ is formulated in Equation (9).

(9) $$J_{k}=h_{1}S_{k}+h_{2}G_{k}$$

The coefficients $h_{1}$ and $h_{2}$ (satisfying $h_{1}+h_{2}=1$) are the weights of the size and gradient terms, respectively. If $J_{k}$ is large, the corresponding group has both a relatively large number of elements and a strong field variation with respect to its neighboring groups; thus, it is regarded as a high-dynamic group and is subdivided by applying *K*-means clustering again. If $J_{k}$ is small, the current grouping is retained. In this work, a threshold $J_{th}$ is used for the decision. A group is subdivided when $J_{k}$ is not smaller than $J_{th}$ and the number of elements in that group is larger than the minimum group size, which is set to four in this paper. The subdivision procedure is repeated until no group satisfies the subdivision condition.

### 2.3. Array Pattern Reconstruction

After obtaining a grouping result with reasonable group size and smooth inter-group field variation, the active element patterns of the non-representative elements are reconstructed from the representative element pattern in each group. Once all element patterns are obtained, the array radiation pattern under different beam-steering directions can be calculated.

For an element located in the same quadrant as its representative element, the element pattern is reconstructed by applying a phase compensation to the representative element pattern. For the *n*th element in the *k*th group, the relative displacement from the representative element is $△r_{n}=\left(\left.△x_{n},△y_{n}\right)\right.$. $E_{rep_{k}}^{\theta }$ and $E_{rep_{k}}^{\phi }$ are θ and ϕ components, respectively, of the far-field patterns $E_{rep_{k}}$ for the *k*th set of representative elements. As formulated in Equations (10) and (11), the reconstructed components $E_{n}^{\theta }$ and $E_{n}^{\phi }$ correspond to the θ and ϕ components of the far-field pattern for the *n*th element.

(10) $$E_{n}^{\theta }(\theta ,\phi )=E_{rep_{k}}^{\theta }(\theta ,\phi )\cdot e^{+jk_{0}(\Delta x_{n}\sin\theta \cos\phi +\Delta y_{n}\sin\theta \sin\phi )}$$

(11) $$E_{n}^{\phi }(\theta ,\phi )=E_{rep_{k}}^{\phi }(\theta ,\phi )\cdot e^{+jk_{0}(\Delta x_{n}\sin\theta \cos\phi +\Delta y_{n}\sin\theta \sin\phi )}$$

By analyzing the radiation patterns of elements across different quadrants, it is demonstrated that deriving the pattern of an element located in a different quadrant from the representative element requires a two-step procedure: mapping the azimuthal angle ϕ and correcting the sign of the polarization vector. The query angle in the representative pattern is mapped to the corresponding mirror angle $\phi^{\prime }$, and the signs of the $E_{rep_{k}}^{\theta }$ and $E_{rep_{k}}^{\phi }$ polarized components are corrected by $\sigma^{\theta }$ and $\sigma^{\phi }$.

(12) $$(\phi^{\prime },\sigma^{\theta },\sigma^{\phi })=\left\{\begin{array}{l} (180^{\circ }-\phi ,-1,+1)\;\;\;\;\;x_{n}\cdot x_{rep}\leq 0,y_{n}\cdot y_{rep}\geq 0\\ (360^{\circ }-\phi ,+1,-1)\;\;\;\;\;x_{n}\cdot x_{rep}\geq 0,y_{n}\cdot y_{rep}\leq 0\\ (180^{\circ }+\phi ,-1,-1)\;\;\;\;\;x_{n}\cdot x_{rep}\geq 0,y_{n}\cdot y_{rep}\geq 0 \end{array}\right.$$

(13) $$E_{n}^{\theta }(\theta ,\phi )=\sigma^{\theta }\cdot E_{rep_{k}}^{\theta }(\theta ,\phi )\cdot e^{+jk_{0}(\Delta x_{n}\sin\theta \cos\phi +\Delta y_{n}\sin\theta \sin\phi )}$$

(14) $$E_{n}^{\phi }(\theta ,\phi )=\sigma^{\phi }\cdot E_{rep_{k}}^{\phi }(\theta ,\phi )\cdot e^{+jk_{0}(\Delta x_{n}\sin\theta \cos\phi +\Delta y_{n}\sin\theta \sin\phi )}$$

When the element to be reconstructed and the representative element are located in different quadrants, their values are given by Equation (12), where $r_{rep}=\left(x_{rep},y_{rep}\right)$ represents the spatial coordinates of the representative element within that group. In this case, the $E_{n}^{\theta }$ and $E_{n}^{\phi }$ components of the far-field radiation pattern for the element to be reconstructed are given by Equation (13) and Equation (14), respectively. After the active element pattern of each array element has been reconstructed, the required phase excitation is applied to synthesize beams pointing to different directions. For a target beam direction $\left(\theta_{s},\phi_{s}\right)$, the excitation weight of the *n*th element is calculated according to Equation (15).

(15) $$w_{n}=e^{-jk_{0}(x_{n}\sin\theta_{s}\cos\phi_{s}+y_{n}\sin\theta_{s}\sin\phi_{s})}$$

The reconstructed array pattern is obtained by coherently summing the phase-weighted element contributions over all array elements, as shown in Equation (16).

(16) $$b_{n}(\theta ,\phi )=\sum_{n=1}^{N}w_{n}\cdot E_{n}(\theta ,\phi )$$

### 2.4. All-On Element Pattern Measurement and Sparse Sampling

To improve the accuracy of the representative element pattern measurement, this work obtains the representative element patterns in the all-on mode [11]. In this mode, all elements are excited simultaneously, thereby providing a measured response that more accurately reflects the actual mutual-coupling environment of the operating array. The measured broadside beam $b_{0}$ under uniform phase excitation is formulated in Equation (17).

(17) $$b_{0}(\theta ,\phi )=\sum_{n=1}^{N}E_{n}(\theta ,\phi )$$

To retrieve the pattern of the *n*th element, an additional measurement is performed by keeping all elements active while setting the phase excitation of the *n*th element to $180^{\circ }$, with the other elements still excited at $0^{\circ }$ phase. The array pattern measured under this condition, denoted as $s_{n}$, is formulated in Equation (18).

(18) $$s_{n}(\theta ,\phi )=\sum_{\begin{array}{l} q=1,\\ q\neq n \end{array}}^{N}E_{q}(\theta ,\phi )-E_{n}(\theta ,\phi )$$

Consequently, by combining Equations (17) and (18), the active element pattern measured in the all-on mode is formulated in Equation (19).

(19) $$E_{n}(\theta ,\phi )=\frac{b_{0}(\theta ,\phi )-s_{n}(\theta ,\phi )}{2}$$

To provide a more comprehensive analysis, two-dimensional pattern similarity and equivalent stray signal (ESS) [23,24] are adopted as evaluation metrics. Pattern similarity assesses agreement over the full two-dimensional angular domain, whereas ESS assesses the discrepancy on the $\phi =\phi_{s}$ cut associated with each steering direction. The gain pattern similarity is computed from the separately power-normalized reference and reconstructed total-field patterns over all Q angular samples:

(20) $$\eta =\left(1-\frac{1}{2}\sum_{q=1}^{Q}\left|\frac{{\left|P_{ref}\left(\Omega_{q}^{O}\right)\right|}^{2}}{\sum_{q^{\prime }=1}^{Q}{\left|P_{ref}\left(\Omega_{q^{\prime }}^{O}\right)\right|}^{2}}-\frac{{\left|P_{reco}\left(\Omega_{q}^{O}\right)\right|}^{2}}{\sum_{q^{\prime }=1}^{Q}{\left|P_{reco}\left(\Omega_{q^{\prime }}^{O}\right)\right|}^{2}}\right|\right)$$

where $P_{ref}$ refers to the reference array radiation pattern, and $P_{reco}$ corresponds to the reconstructed array radiation pattern. The symbol $\Omega_{q}^{o}$ denotes the *q*th observation direction, while Q defines a 2D angular domain with $\theta \in [0^{\circ },90^{\circ }]$ and $\phi \in [0^{\circ },360^{\circ }]$. A larger similarity value indicates closer agreement. ESS evaluates the error on the $\phi =\phi_{s}$ cut associated with the steering direction; a lower ESS indicates smaller discrepancy.

Furthermore, the angular sampling grid Ω=(θ,ϕ) can be sparsified during the element pattern measurement. Because the element pattern in conventional antenna arrays typically exhibits smooth spatial variations, it is highly feasible to employ an appropriately sparse sampling interval. The element pattern can then be reconstructed via interpolation, thereby further reducing the total measurement time.

In summary, the proposed method first removes the common phase factor from the broadside far-field array pattern and constructs a feature matrix that combines element position and corrected phase information. *K*-means clustering is then refined by a composite criterion that accounts for both group size and inter-group field variation, so that high-dynamic regions are subdivided while slowly varying regions are kept compact. The active element patterns of all array elements are reconstructed from the representative elements through phase compensation and symmetry mapping, and the final beam patterns are synthesized by applying the required steering weights. Together with the all-on representative-element measurement and optional sparse angular sampling, the method reduces the number of required element-pattern measurements while preserving the physical coupling environment and the accuracy of multi-beam testing of phased arrays.

## 3. Results

### 3.1. Simulation Validation

**Figure 1:**
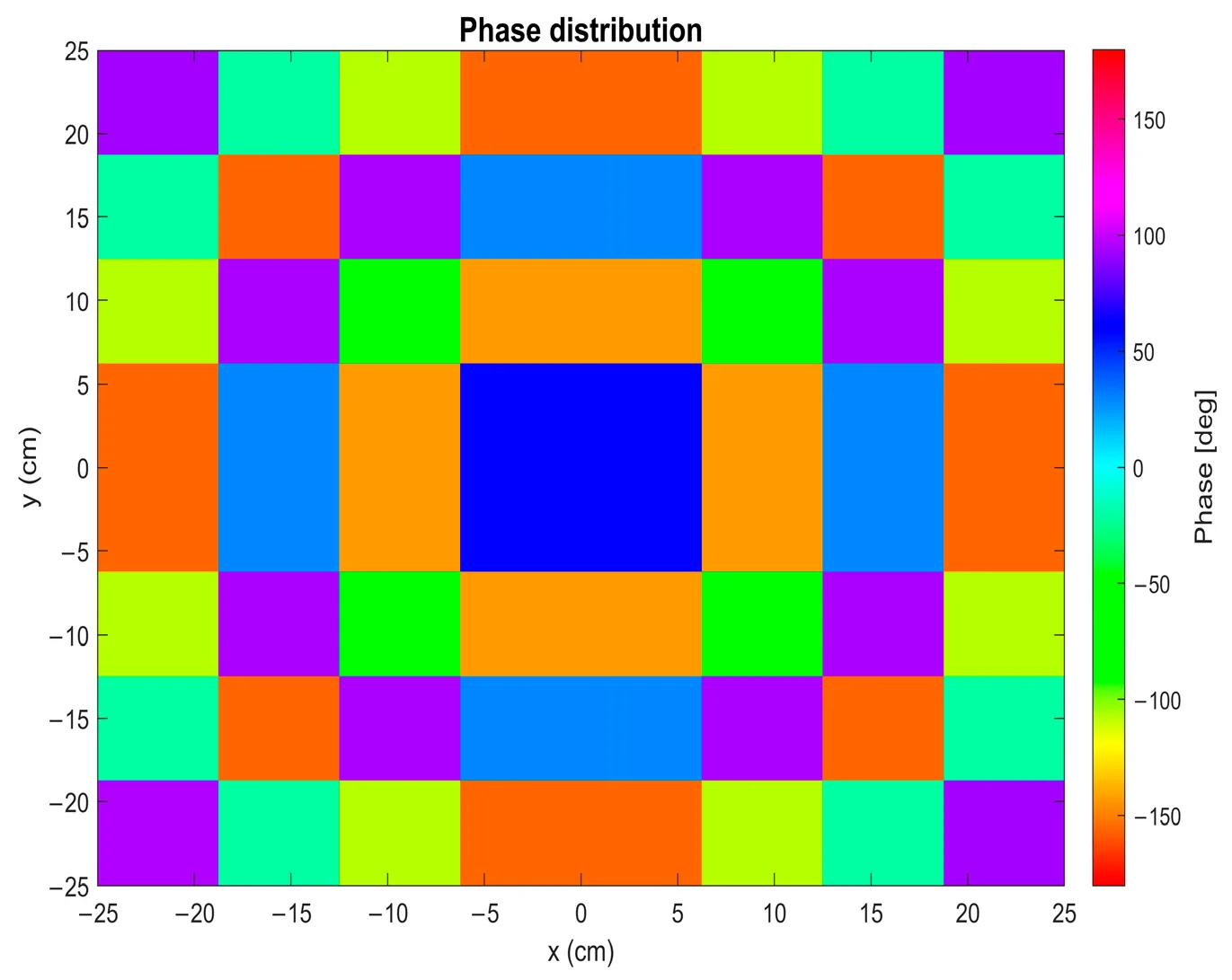
Phase distribution at the element positions after extraction of the common phase factor for the 8 × 8 dipole array.

The proposed adaptive grouping strategy is first evaluated using an 8×8 dipole array operating at 2.4 GHz with half-wavelength element spacing. After removal of the common phase factor, the residual phase distribution at the element positions, shown in Figure 1, captures the remaining element-level variation and serves as an input to the grouping process.

**Figure 2:**
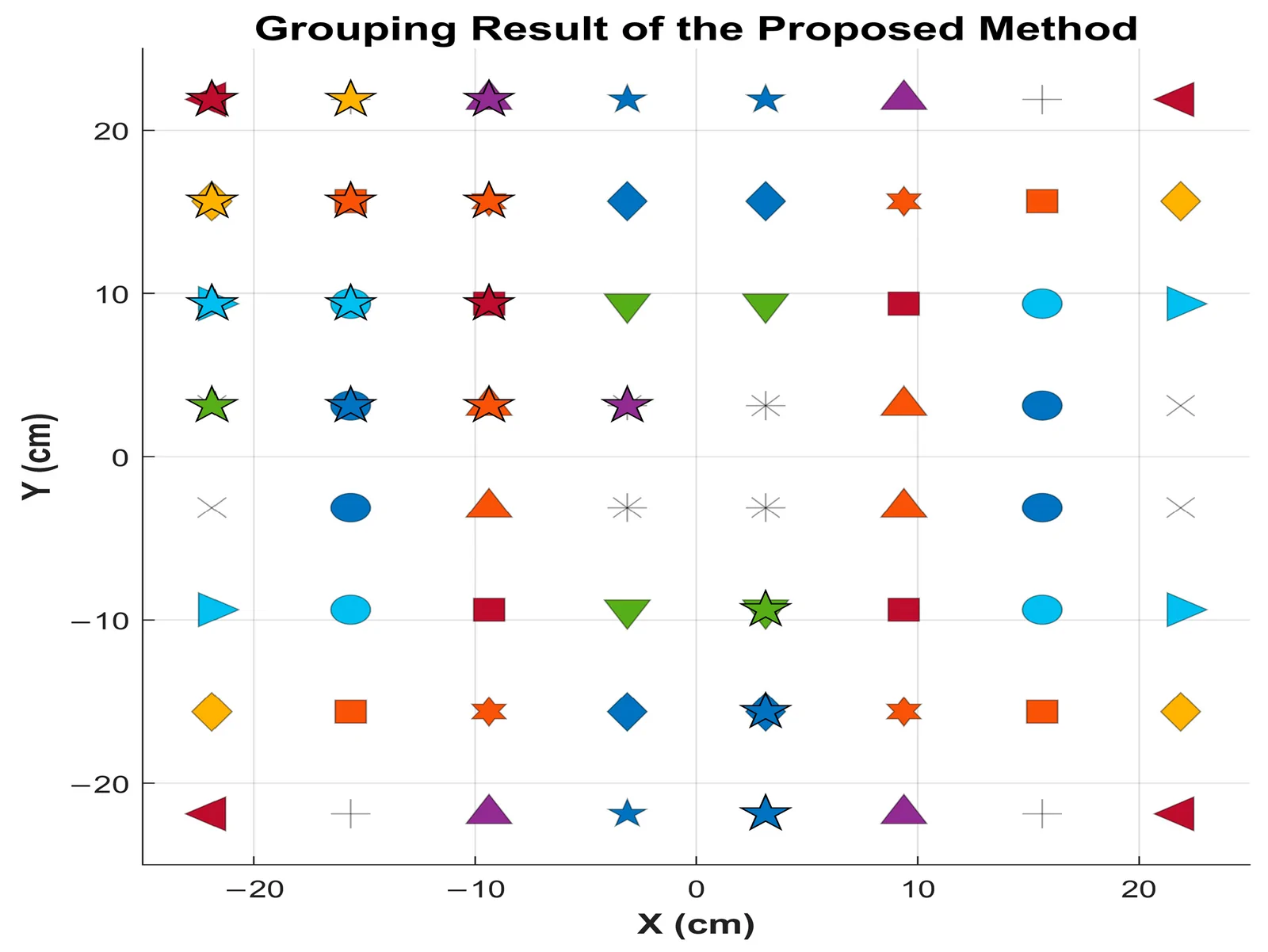
Adaptive clustering result for the 8 × 8 dipole array. Elements with identical color and marker shape are assigned to the same group, and the star marker indicates the representative element.

**Figure 3:**
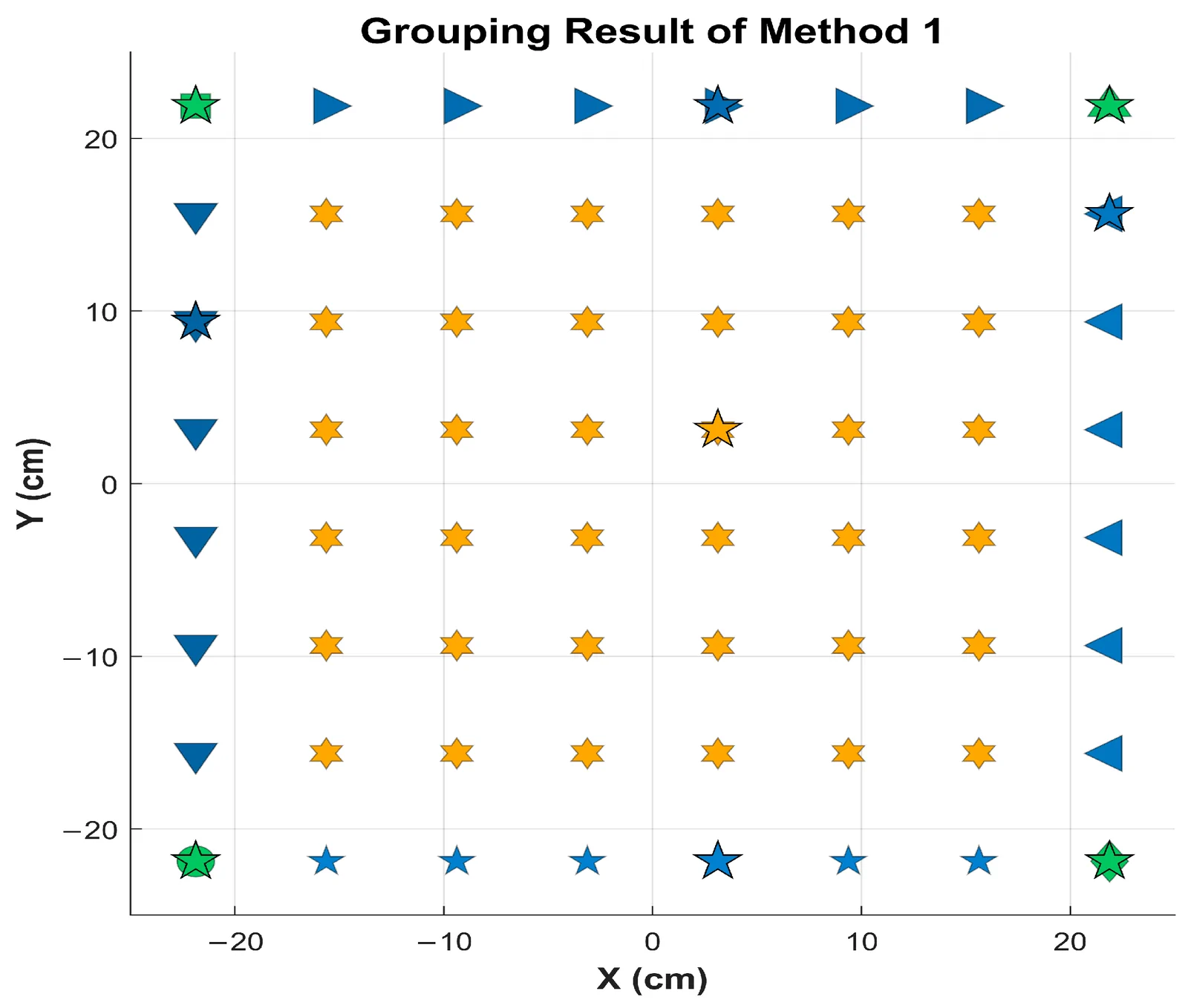
Grouping result of Method 1.

**Figure 4:**
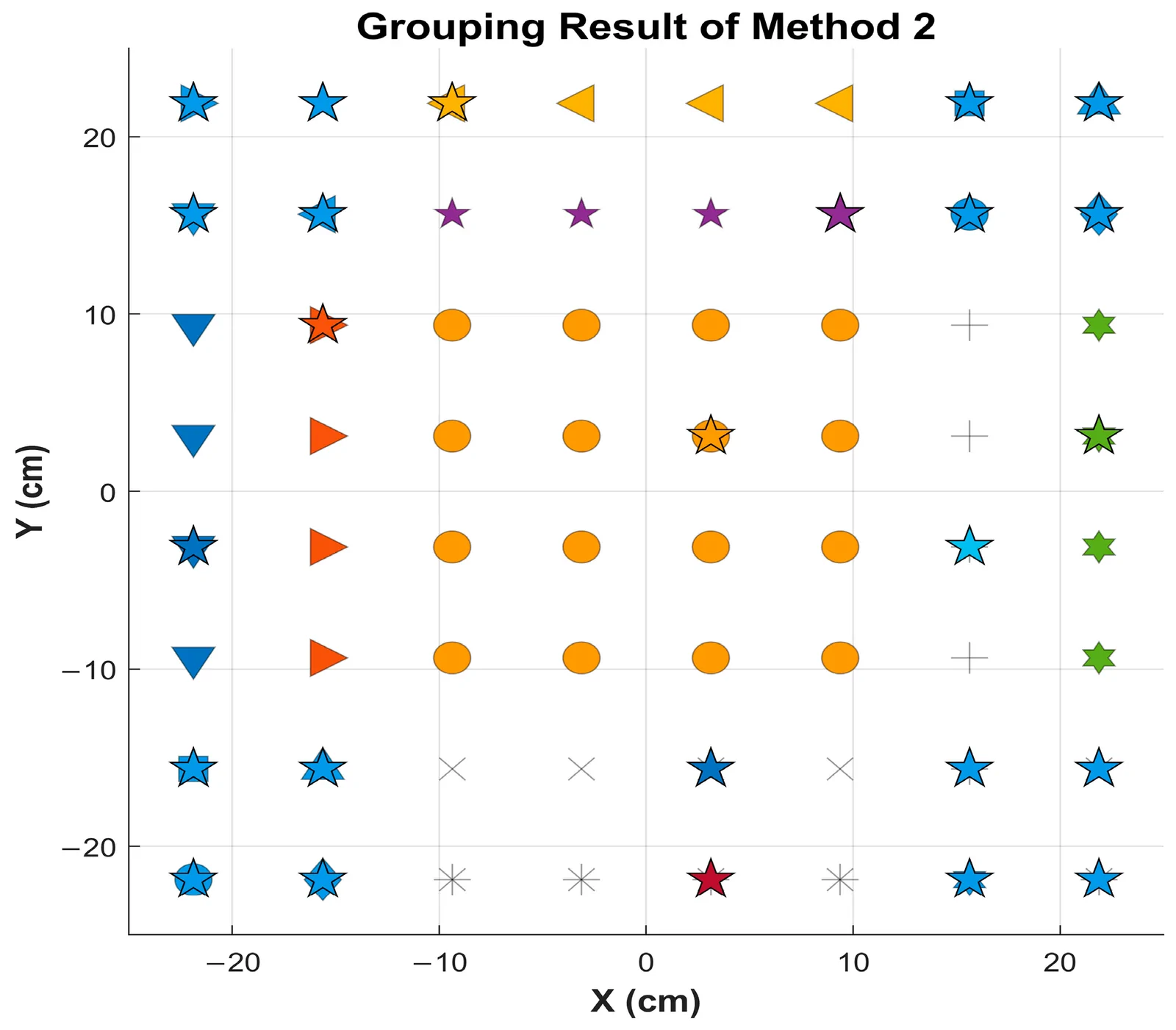
Grouping result of Method 2.

The electric field values at the array-element positions and the corresponding geometric coordinates are then assembled as the feature matrix for adaptive K-means clustering. The classification result is shown in Figure 2, where elements of the same color and shape belong to the same group and the star marker indicates the group representative. For comparison, the 3×3 and 5×5 grouping schemes from [17] are used as Grouping Method 1 and Grouping Method 2, respectively, with their configurations depicted in Figure 3 and Figure 4. For the remaining elements in the same group, the active element patterns are reconstructed from the representative pattern through phase compensation, which yields the complete set of element patterns for the full array.

To assess the effect of grouping on beam synthesis, the element patterns reconstructed by different strategies are excited with the corresponding amplitude and phase weights. Nine beam-steering cases are considered: [θ,ϕ] = $\left[0^{\circ },0^{\circ }\right]$, $\left[0^{\circ },10^{\circ }\right]$, $\left[0^{\circ },20^{\circ }\right]$, $\left[15^{\circ },0^{\circ }\right]$, $\left[15^{\circ },20^{\circ }\right]$, $\left[30^{\circ },0^{\circ }\right]$, $\left[30^{\circ },10^{\circ }\right]$ and $\left[30^{\circ },20^{\circ }\right]$. The reference pattern is calculated from the full-wave simulated active element patterns of all 64 elements. The comparison also includes the proposed method combined with a $10^{\circ }$ sparse sampling reconstruction of the representative-element patterns, as well as two existing grouping strategies: Grouping Method 1, corresponding to the 3×3 grouping scheme, and Grouping Method 2, corresponding to the 5×5 grouping scheme.

**Figure 5:**
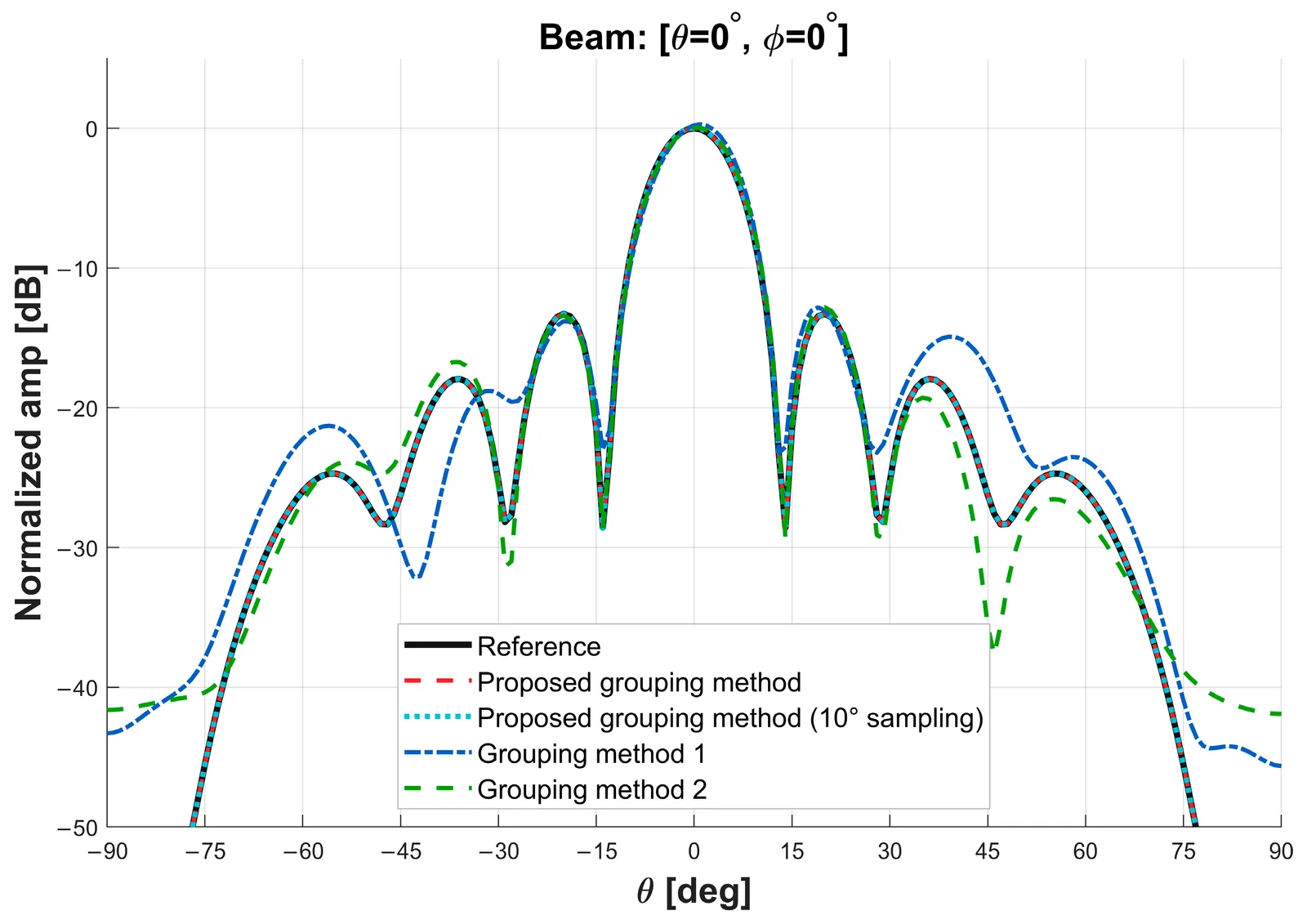
Simulated array patterns for Beam 1 $\left(\theta =0^{\circ },\phi =0^{\circ }\right)$ $\phi =0^{\circ }$ cut obtained using different grouping strategies.

**Figure 6:**
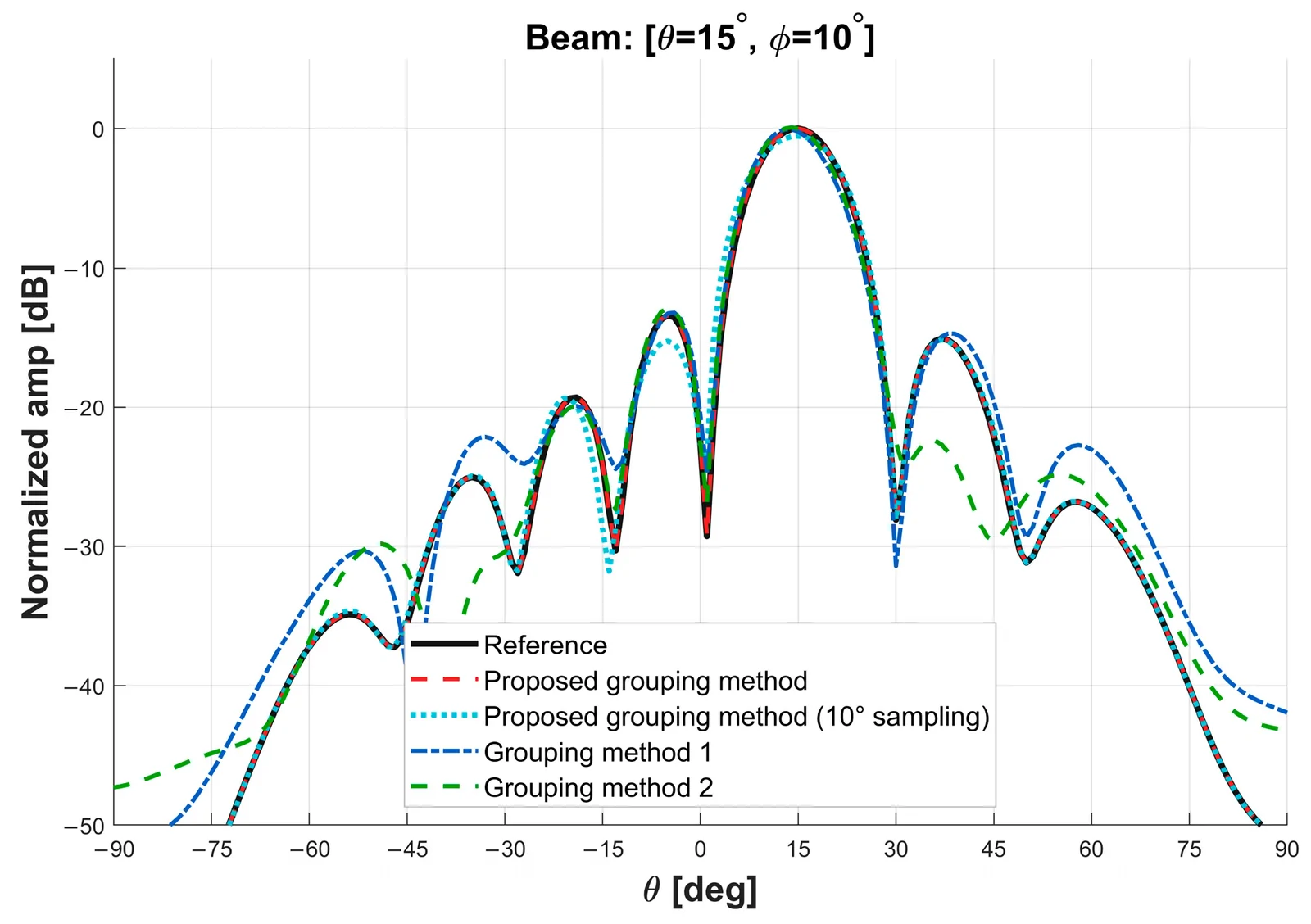
Simulated array patterns for Beam 2 $\left(\theta =15^{\circ },\phi =10^{\circ }\right)$ $\phi =10^{\circ }$ cut obtained using different grouping strategies.

**Figure 7:**
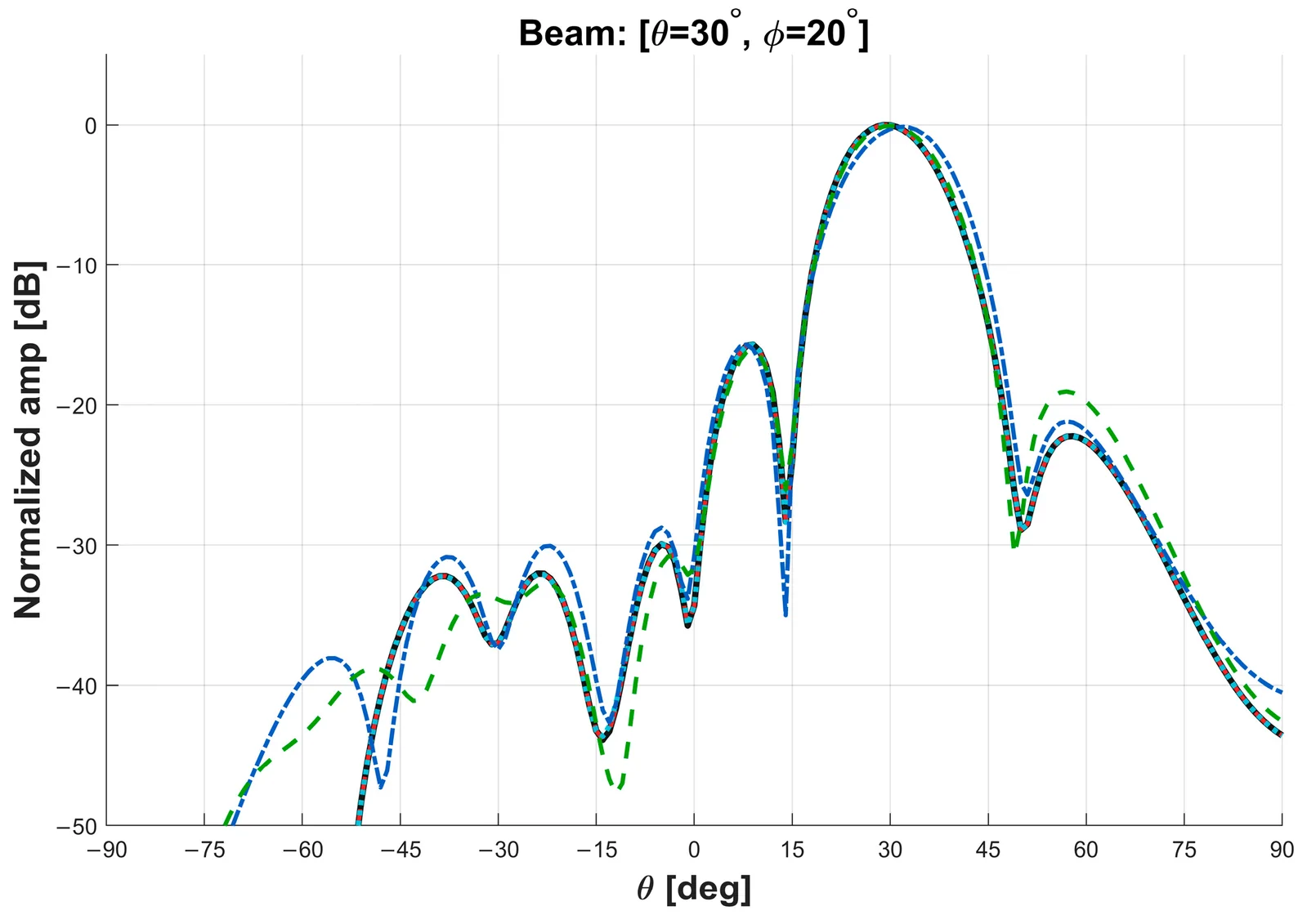
Simulated array patterns for Beam 3 $\left(\theta =30^{\circ },\phi =20^{\circ }\right)$ $\phi =20^{\circ }$ cut obtained using different grouping strategies.

**Figure 8:**
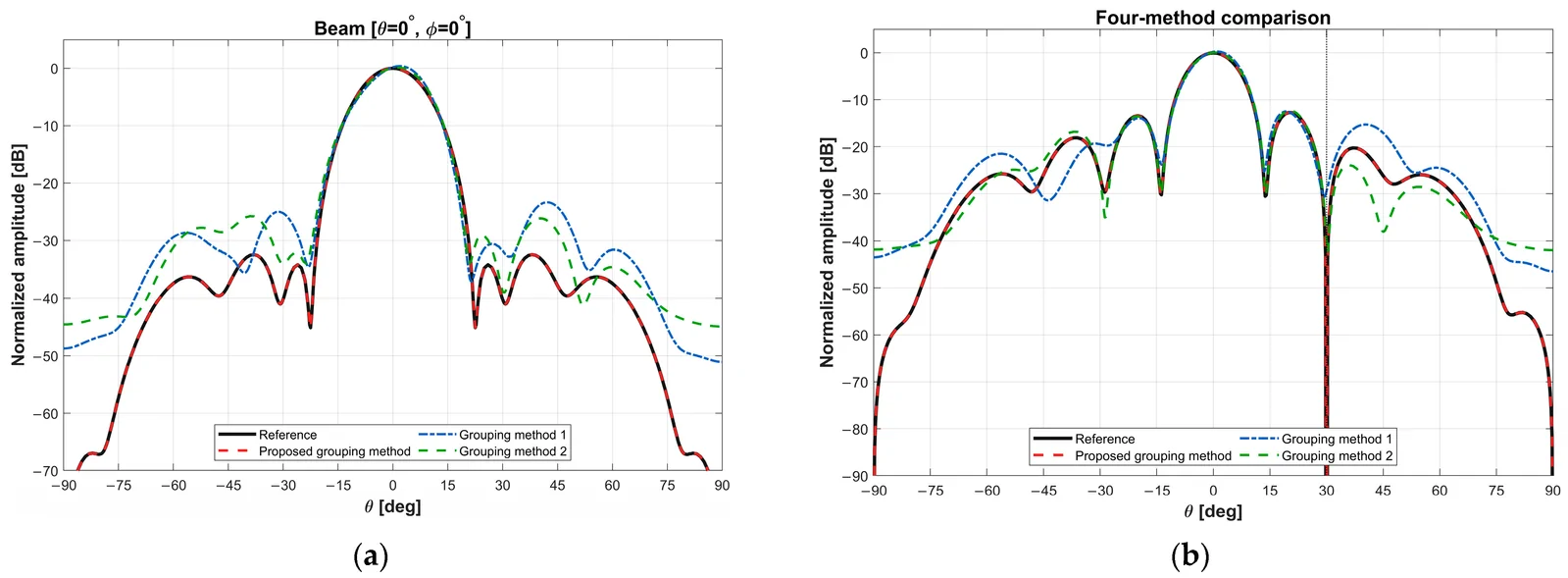
The tapering and null formation results for beam $\left(\theta =0^{\circ },\phi =0^{\circ }\right)$ $\phi =0^{\circ }$ cut. (**a**) Tapering results. (**b**) Null formation at $\theta_{i}=30^{\circ }$.

Figure 5, Figure 6 and Figure 7 show that the proposed grouping method reproduces the reference array pattern with high consistency for all three steering angles. The reconstructed patterns obtained by the proposed method agree well with the reference patterns for all simulated beam directions. The major lobe positions are accurately maintained, and the dominant nulls and sidelobe trends are generally reproduced. The $10^{\circ }$ sparse-sampling result also remains close to the reference, showing only minor deviations. Compared with the proposed method, the fixed grouping methods show larger differences in several minor lobes and null regions, especially for the scanned beams. Quantitatively, the 8 × 8 simulation requires 16 representative elements instead of 25, corresponding to a 36% reduction in representative-element measurements, while retaining the main-beam position and improving several sidelobe-null regions. Furthermore, to present additional illustrative pattern results and investigations, two further cases are considered: Taylor amplitude tapering and linearly constrained single-null formation. The simulation results are summarized in Figure 8a,b. Figure 8a shows the corresponding results obtained with a two-dimensional Taylor amplitude taper designed for a −30 dB peak-sidelobe target at the steering direction $\left[0^{\circ },0^{\circ }\right]$. Figure 8b shows the result of imposing a single null at $\theta_{i}=30^{\circ }$.

**Figure 9:**
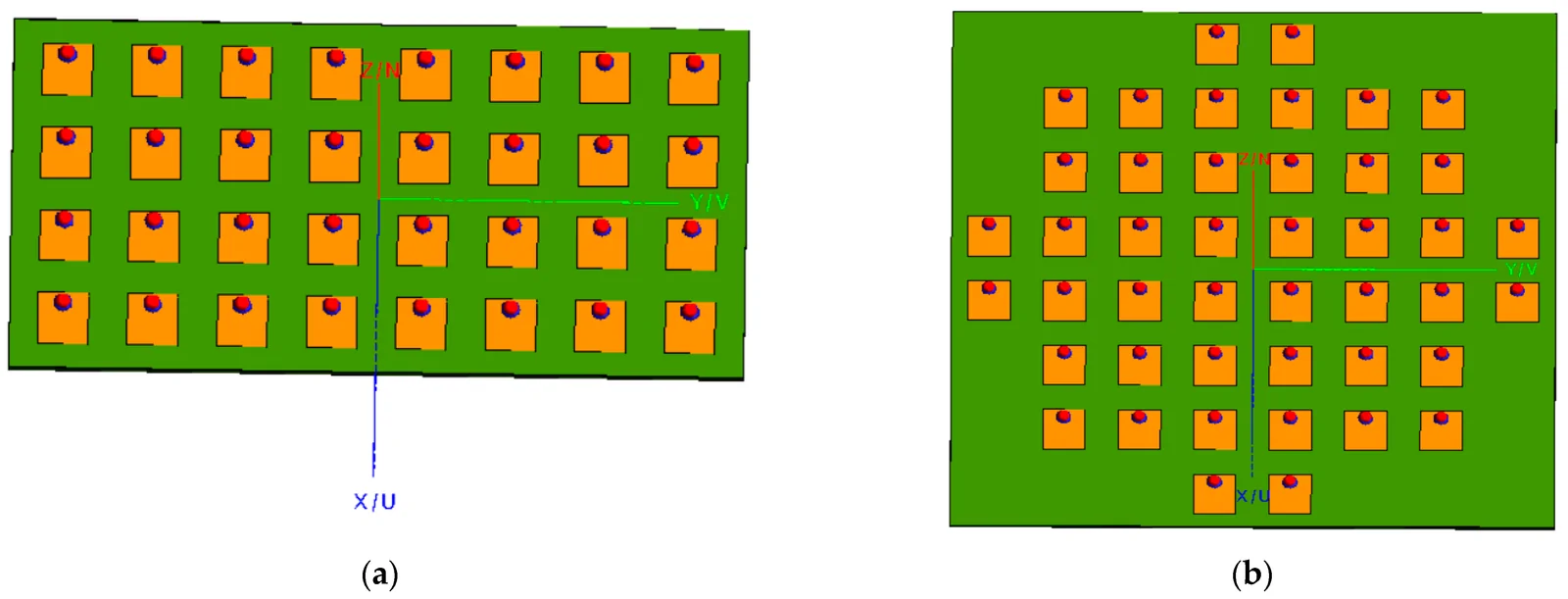
Geometries of the patch antenna arrays: (**a**) 4×8 patch antenna array. (**b**) Irregular 44-element patch antenna array.

**Figure 10:**
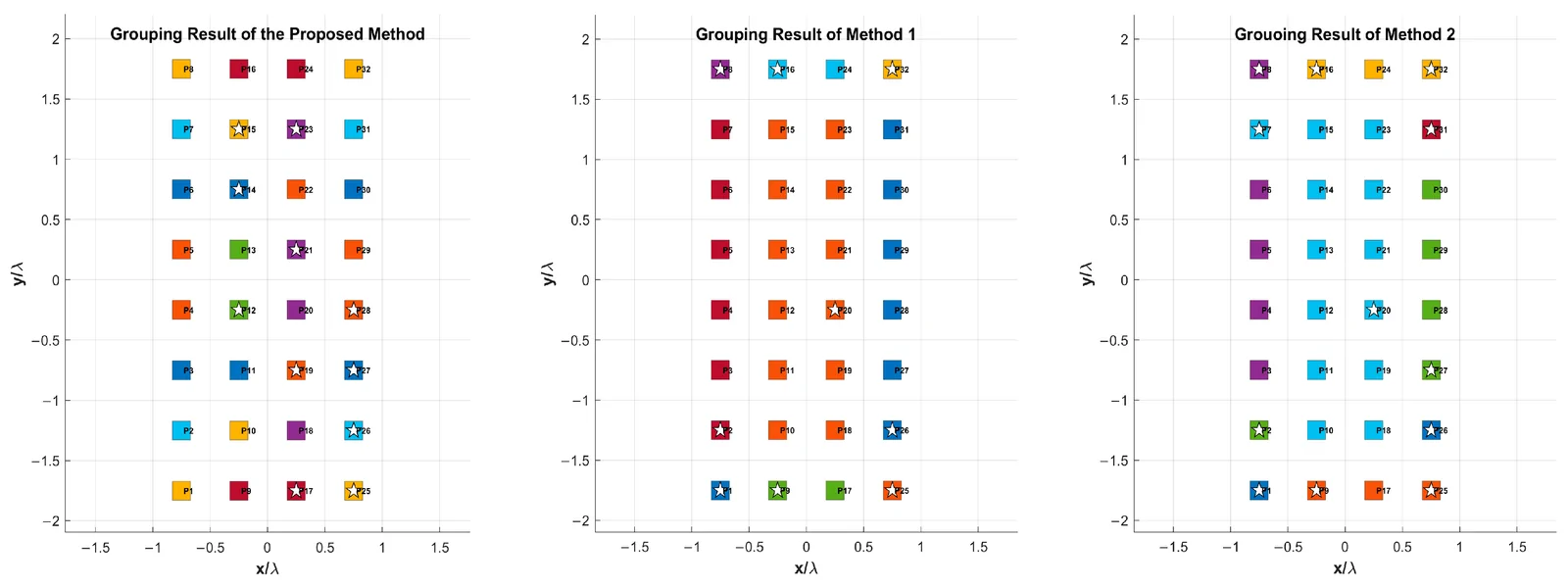
Grouping results for the 4×8 patch antenna array with 11 representative elements for the proposed method, 9 for Grouping Method 1, and 12 for Grouping Method 2.

**Figure 11:**
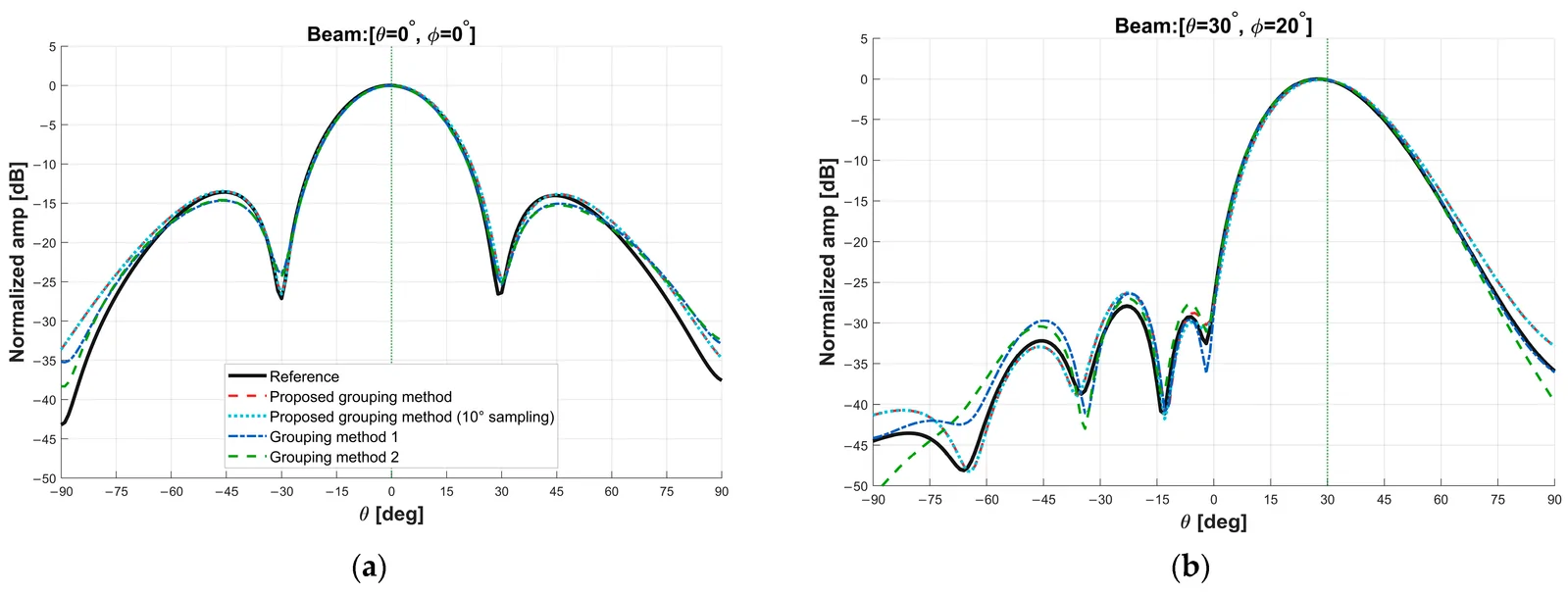
Simulated 4×8 patch antenna array patterns for beam $\left(\theta =0^{\circ },\phi =0^{\circ }\right)$ and $\left(\theta =30^{\circ },\phi =20^{\circ }\right)$, obtained using different grouping strategies. (**a**) Beam $\left(\theta =0^{\circ },\phi =0^{\circ }\right)$ $\phi =0^{\circ }$ cut. (**b**) Beam $\left(\theta =30^{\circ },\phi =20^{\circ }\right)$ $\phi =20^{\circ }$ cut.

**Figure 12:**
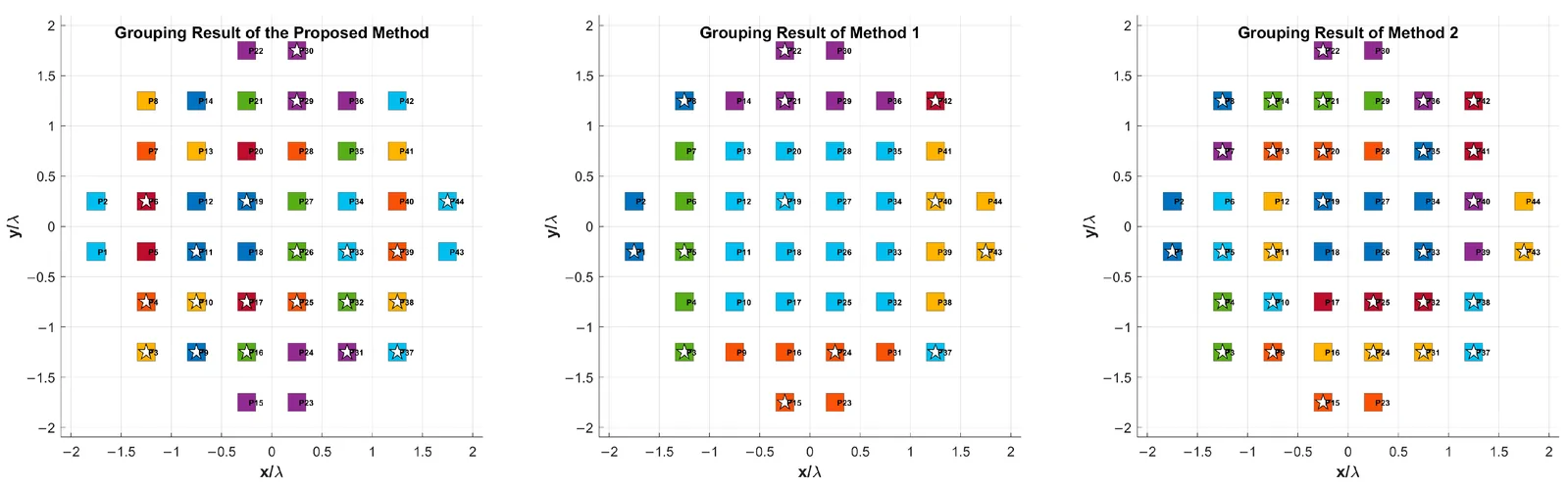
Grouping results for the irregular 44-element patch antenna array with 20 representative elements for the proposed method, 13 for Grouping Method 1, and 29 for Grouping Method 2.

**Figure 13:**
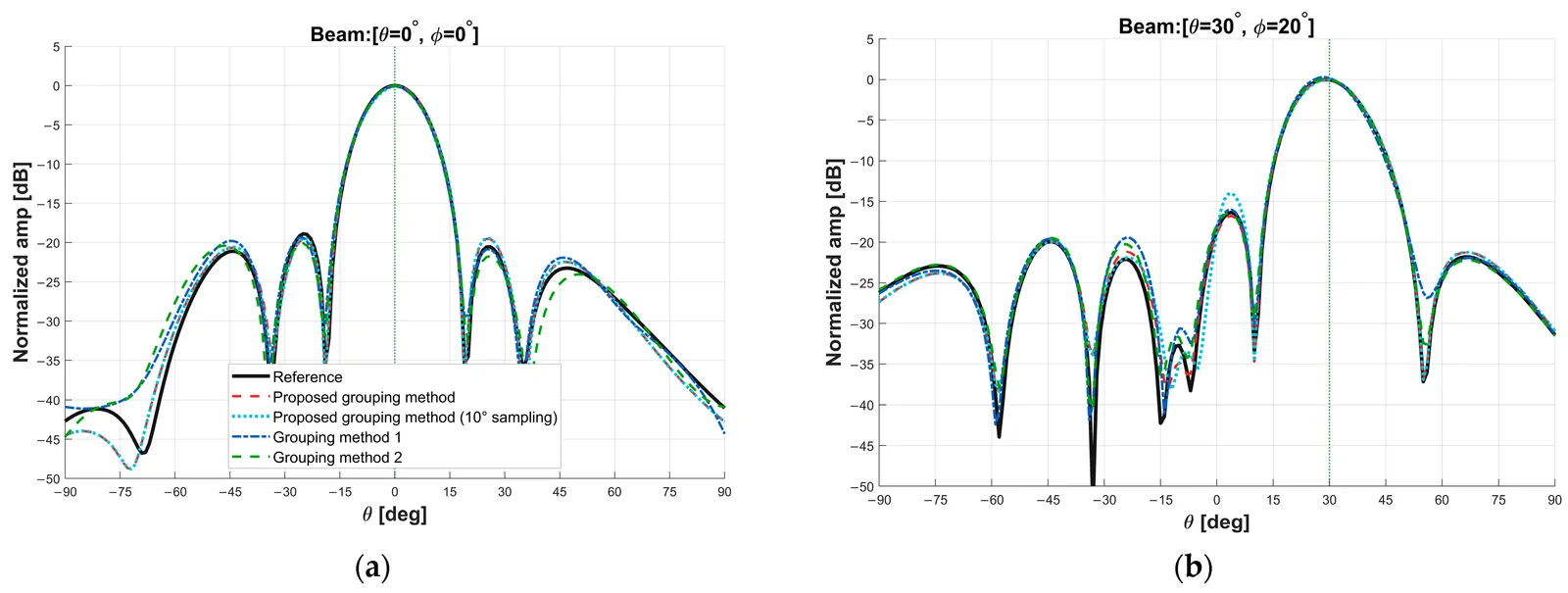
Simulated irregular 44-element patch antenna array patterns for beams $\left(\theta =0^{\circ },\phi =0^{\circ }\right)$ and $\left(\theta =30^{\circ },\phi =20^{\circ }\right)$, obtained using different grouping strategies. (**a**) Beam $\left(\theta =0^{\circ },\phi =0^{\circ }\right)$ $\phi =0^{\circ }$ cut. (**b**) Beam $\left(\theta =30^{\circ },\phi =20^{\circ }\right)$ $\phi =20^{\circ }$ cut.

To verify the applicability of the proposed grouping method to arrays with irregular geometries, different array sizes, and different antenna types, a 4×8 patch antenna array and an irregular 44-element patch antenna array were simulated. Figure 9 shows the geometries of the 4×8 patch antenna array and the irregular 44-element array. Figure 10 presents the grouping results for the 4×8 array, and Figure 11 compares the corresponding array patterns. Figure 12 and Figure 13 show the grouping results and the array pattern comparison for the irregular 44-element patch array, respectively.

### 3.2. Experimental Validation Using a 4 × 4 Millimeter-Wave Array

**Figure 14:**
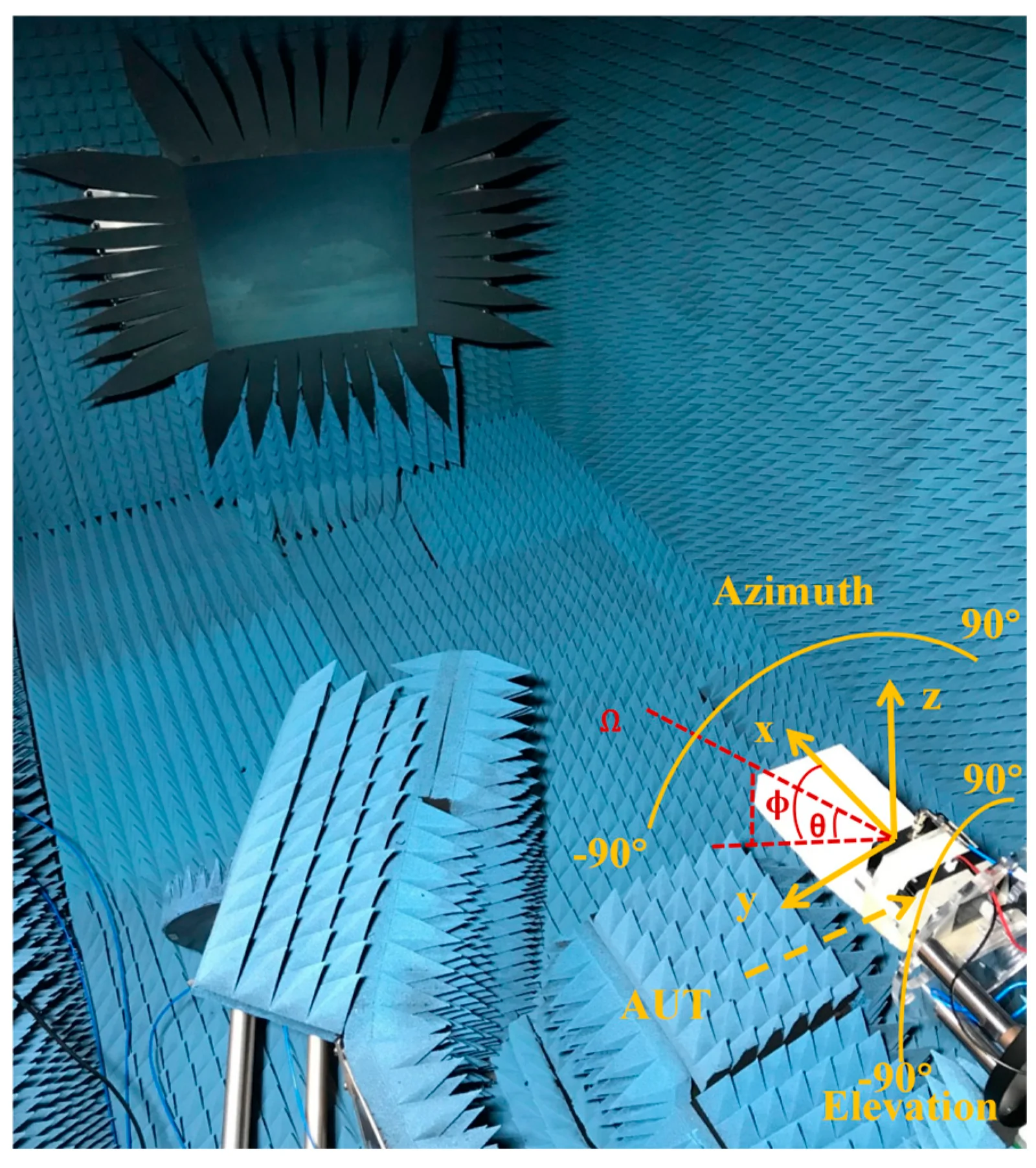
Measurement setup for the 4 × 4 mmWave antenna array operating at 28 GHz.

The proposed method is further validated using measured data from a 4×4 mmWave antenna array operating at 28 GHz. The detailed array dimensions are described in [25], where the prototype photograph shows the antenna elements concealed beneath the product nameplate label. The measurement configuration is shown in Figure 14. This experimental case provides an additional test under practical mutual-coupling and measurement conditions, where active element patterns are affected by fabrication tolerances, fixture effects, and the surrounding measurement environment.

**Figure 15:**
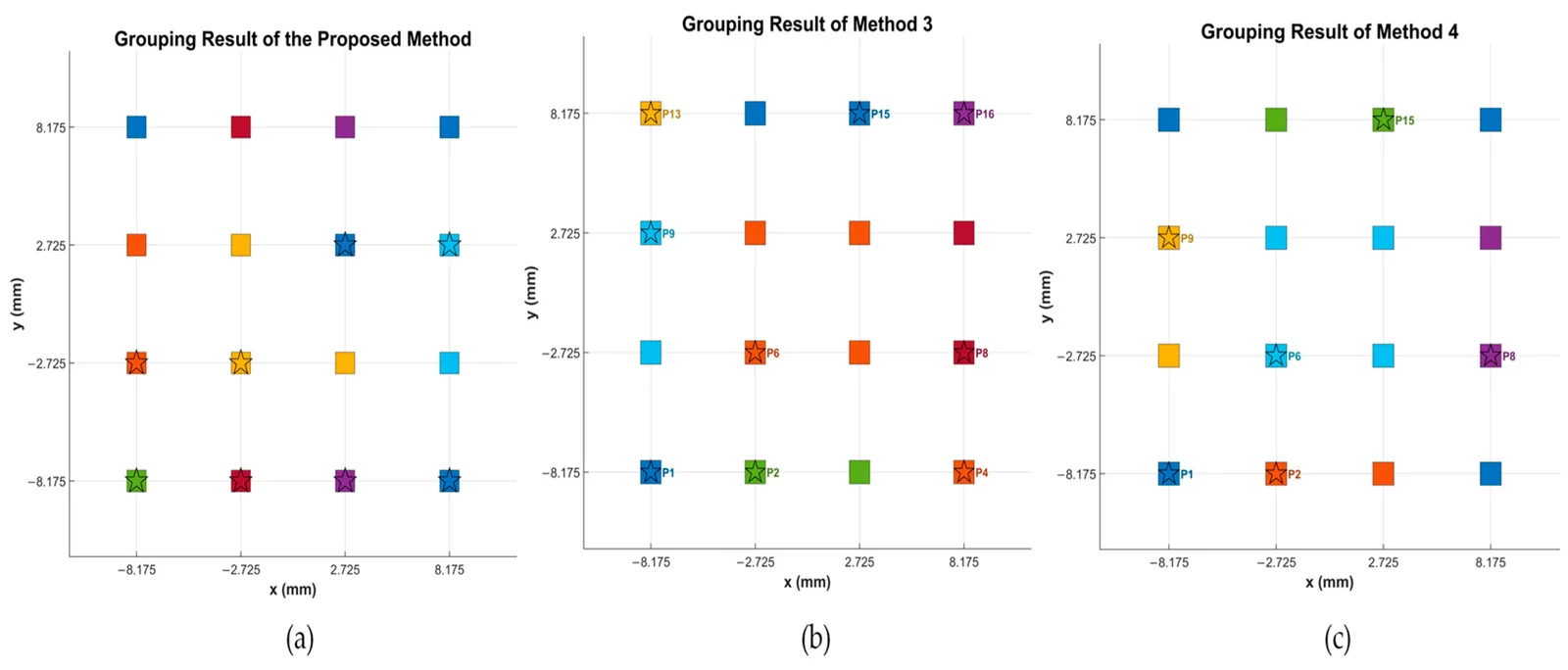
Grouping results for the measured 4 × 4 mmWave array: (**a**) proposed grouping method; (**b**) Grouping Method 3; (**c**) Grouping Method 4.

**Figure 16:**
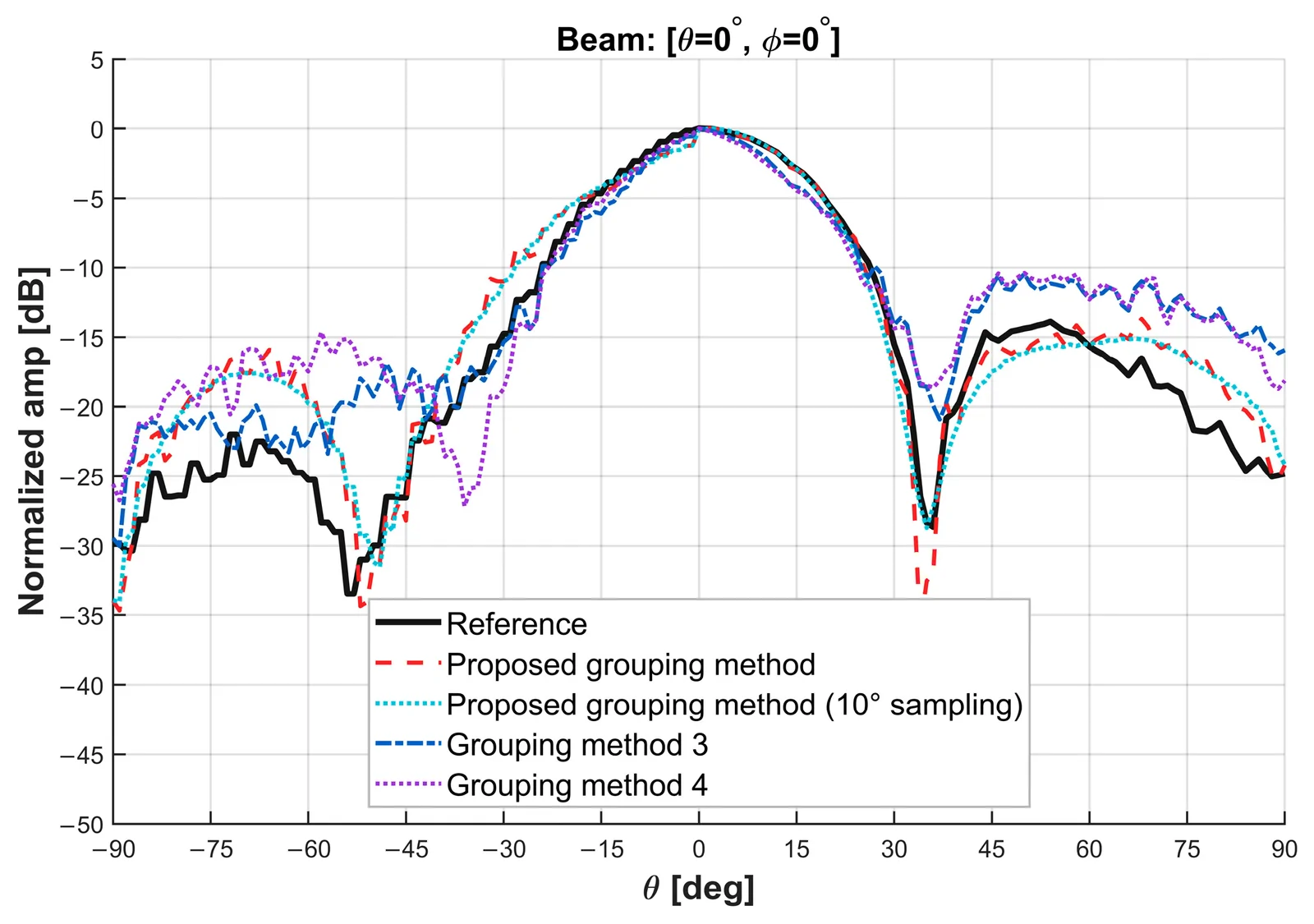
Measured array patterns for Beam 1 $\left(\theta =0^{\circ },\phi =0^{\circ }\right)$ $\phi =0^{\circ }$ cut obtained using different grouping strategies.

**Figure 17:**
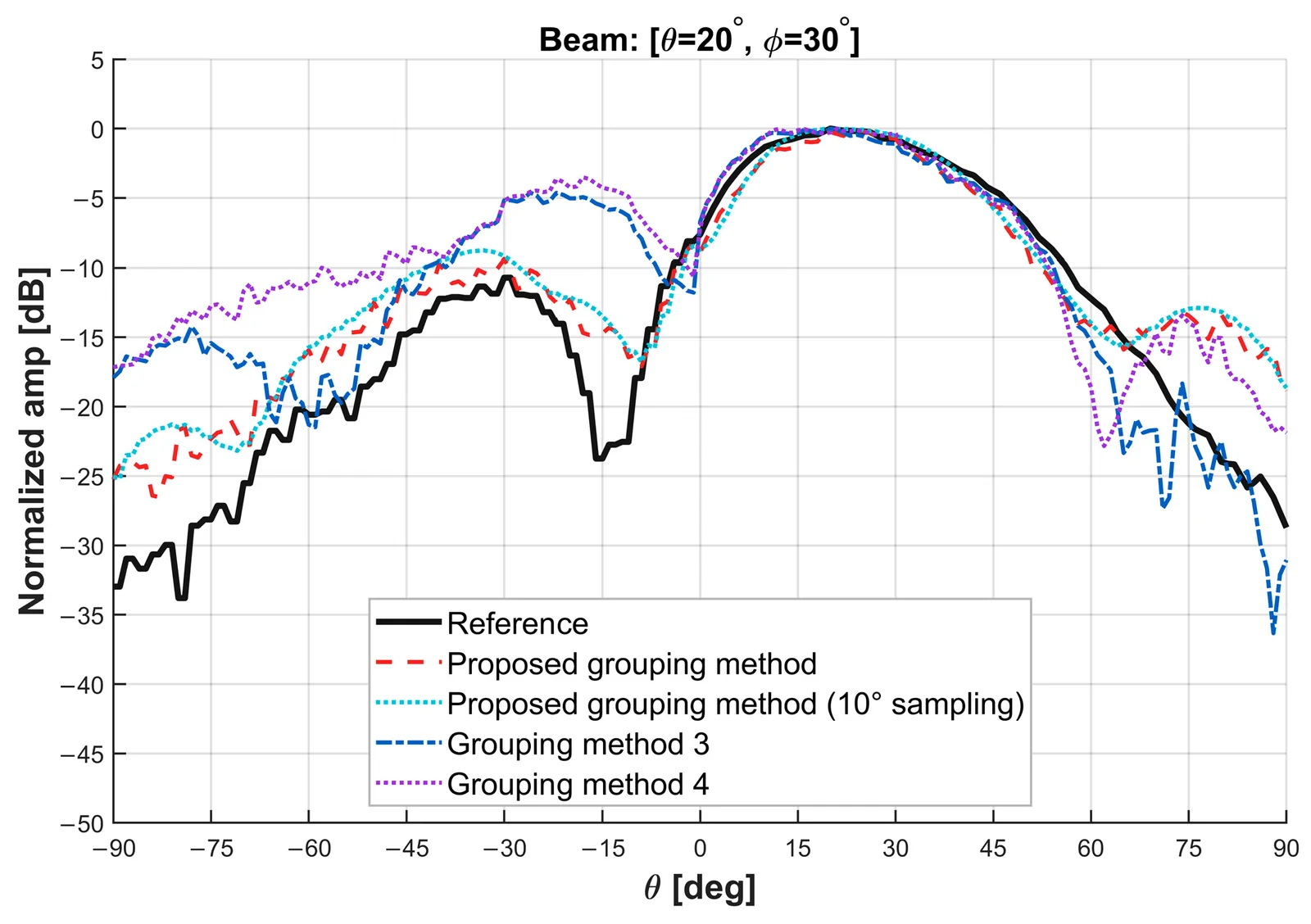
Measured array patterns for Beam 2 $\left(\theta =20^{\circ },\phi =30^{\circ }\right)$ $\phi =30^{\circ }$ cut obtained using different grouping strategies.

**Figure 18:**
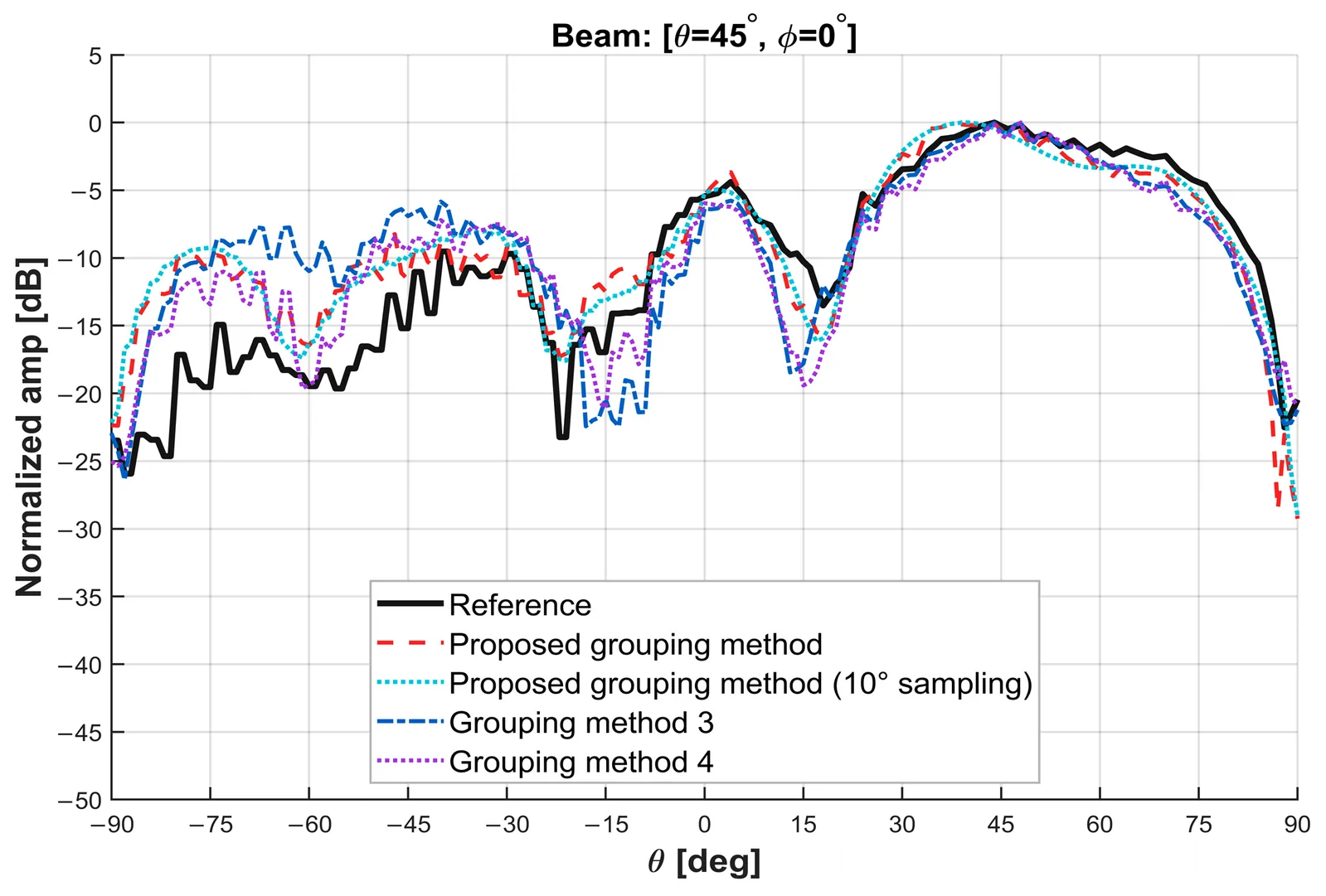
Measured array patterns for Beam 3 $\left(\theta =45^{\circ },\phi =0^{\circ }\right)$ $\phi =0^{\circ }$ cut obtained using different grouping strategies.

Figure 15 compares the grouping result produced by the proposed method with two coupling-environment-based grouping strategies. Grouping Method 3 treats the four corner elements as four independent groups, whereas Grouping Method 4 assigns the four corner elements to the same group. The proposed method forms the groups directly from the extracted features and the element coordinates, allowing the representative elements to be selected according to the measured array response rather than by a manually prescribed corner or edge rule. The measured active element patterns reconstructed by these methods are then used for beam synthesis. Five beam-steering cases are considered: [θ,ϕ] = $\left[0^{\circ },0^{\circ }\right]$, $\left[20^{\circ },0^{\circ }\right]$, $\left[20^{\circ },30^{\circ }\right]$, $\left[30^{\circ },0^{\circ }\right]$, and $\left[45^{\circ },0^{\circ }\right]$. Three steering cases are evaluated in Figure 16, Figure 17 and Figure 18: Beam 1 with $\theta =0^{\circ }$ and $\phi =0^{\circ }$, Beam 2 with $\theta =20^{\circ }$ and $\phi =30^{\circ }$, and Beam 3 with $\theta =45^{\circ }$ and $\phi =0^{\circ }$. The reference pattern is calculated from the measured element patterns of all 16 elements. The proposed grouping method is also evaluated together with the $10^{\circ }$ sparse sampling reconstruction of the representative-element patterns.

To better highlight the advantages of the proposed approach, a comprehensive comparison with relevant methods reported in the literature is presented. For 8×8 dipole antenna array, Table 1 compares the reconstruction results obtained using the proposed method, the proposed method with a $10^{\circ }$ sampling interval, the 3×3 grouping methods in [14,15,16,17], and the 5×5 grouping method in [17] for nine beam-steering directions (i.e., [θ,ϕ] = $\left[0^{\circ },0^{\circ }\right]$, $\left[0^{\circ },10^{\circ }\right]$, $\left[0^{\circ },20^{\circ }\right]$, $\left[15^{\circ },0^{\circ }\right]$, $\left[15^{\circ },20^{\circ }\right]$, $\left[30^{\circ },0^{\circ }\right]$, $\left[30^{\circ },10^{\circ }\right]$, $\left[30^{\circ },20^{\circ }\right]$). The comparison considers the number of antenna elements under test, the mean values of the maximum and root-mean-square (rms) ESS over the nine beams, the mean 2D radiation-pattern similarity, and the approach used to acquire the element radiation patterns. Table 2 further presents the corresponding comparison results obtained from measurements of a 4×4 mmWave phased array.

## 4. Discussion

The results demonstrate that extracting the common phase factor is a critical step before feature-based grouping. As reported in [22], when the common phase factor is not removed, the phase pattern of each antenna element varies rapidly, which makes it difficult to identify stable and physically meaningful similarity information. In contrast, after the common phase factor is extracted, the phase distribution at the element positions becomes suitable for clustering, as shown in Figure 1. Therefore, the proposed method does not require manual judgment of active element patterns similarity. Instead, the phase distribution and the geometric coordinates of the array elements are used as the feature matrix and are directly processed by the adaptive classification algorithm. This observation explains why the proposed method should be interpreted as field-informed clustering rather than geometry-only grouping: the geometry features provide a physically meaningful prior, whereas the corrected phase and gradient-based subdivision respond to actual array-response variation.

The representative element in each group is selected as the element whose feature vector is closest to the mean feature vector of that group. This criterion makes the representative element consistent with the local statistical behavior of the group, rather than depending on a predefined position. This is different from the 5×5 selection strategy used in [14,15,16,17], where the eight corner elements located on the outer and inner rings are assigned to independent groups. Such a strategy increases the number of active element patterns that must be measured. By contrast, the proposed grouping result assigns the four outer-corner elements to one group and the four inner-corner elements to another group.

Another important distinction is the measurement mode used to obtain the representative active element patterns. In the compared method, the element patterns are measured under an on–off condition. In this work, the representative element patterns are obtained under the all-on condition, whose advantages have been discussed in [18]. This measurement mode better preserves the operating environment of the full array. Furthermore, the use of $10^{\circ }$ sparse angular sampling further reduces the measurement burden.

For the 8×8 dipole array, Figure 5, Figure 6 and Figure 7 compare the array patterns reconstructed using different grouping methods under three beam-steering directions. For the broadside beam at $\left[\theta =0^{\circ },\phi =0^{\circ }\right]$, the pattern reconstructed using the proposed method agrees more closely with the reference pattern than those obtained using the other grouping methods. In particular, compared with Grouping Method 2, the proposed method provides a more accurate reconstruction of sidelobe and null details using only 16 representative elements. Similar improvements are observed for the other two beam-steering directions. Moreover, the reconstruction accuracy of the proposed method with $10^{\circ }$ sampling does not decrease substantially, indicating the potential for further improvement in measurement efficiency. Figure 8a shows that, for the broadside beam, the peak-sidelobe levels reconstructed using Grouping Methods 1 and 2 remain slightly above the design target of −30 dB, whereas that reconstructed using the proposed method remains below −30 dB. Figure 8b presents the pattern for a main beam directed toward $\left[0^{\circ },0^{\circ }\right]$ with a target null at $\theta_{i}=30^{\circ }$. A distinct null is formed in the specified interference direction while the main-beam direction is essentially preserved. The patterns reconstructed using the proposed method closely follow the reference patterns, with the null levels in the specified directions remaining below −40 dB. Figure 10 and Figure 11 present the results for the 4×8 patch array. The proposed method selects 11 representative elements, compared with 9 and 12 elements selected by the two comparison methods. It accurately reconstructs the main lobe and sidelobes while reducing reconstruction errors. A clear improvement is observed in the corresponding pattern cut. Figure 12 and Figure 13 present the results for the irregular patch array. The proposed method requires 20 representative elements, whereas the two comparison methods require 13 and 29 elements, respectively. These results demonstrate that the proposed method achieves a better balance between reconstruction accuracy and measurement efficiency.

For the measured data, Figure 16, Figure 17 and Figure 18 compare the reconstructed array patterns under three beam-steering directions. The proposed method provides more accurate reconstruction of fine pattern features, particularly the null depths. The quantitative comparisons in Table 1 and Table 2 further show that the proposed method uses 16 representative elements for the 8×8 array, fewer than the 25 elements required by the 5×5 grouping strategy, while achieving higher pattern similarity and a lower ESS value. The proposed method with $10^{\circ }$ sampling maintains a comparable level of reconstruction quality while reducing the required angular sampling. Unlike the compared on–off strategies, both variants of the proposed method operate in the all-on mode, which more closely represents the actual operating state of a phased array. Previous measurements on a 4×4 mmWave phased array have also shown that this mode reduces the amplitude and phase errors associated with element control [18]. For the measured array, the proposed method selects 8 of the 16 array elements as representative elements and achieves a higher mean pattern similarity than the previously reported 3×3 grouping method.

## 5. Conclusions

This paper presented an adaptive grouping method for efficient multi-beam testing of phased arrays. The proposed method uses the *K*-means unsupervised learning algorithm to classify array elements by constructing a feature matrix from the element position information and the broadside phase distribution. A composite evaluation function, which accounts for both the number of elements in each group and the degree of electric-field variation, is introduced to identify groups located in high-dynamic field regions and further subdivide them. This strategy enables effective array-element classification and automatic representative-element selection.

By considering the pattern similarity of corner elements, the proposed method reduces the number of representative elements required for reconstruction. For the 8×8 dipole array, multi-beam array patterns are reconstructed using 16 representative elements, fewer than the 25 elements required by the 5×5 grouping strategy in the compared method, while improving the accuracy of some minor lobe regions. In addition, the representative element patterns are obtained in the all-on mode, which is more consistent with the practical operating state of phased array elements than the conventional on–off measurement mode. Sparse angular sampling is also incorporated to further reduce measurement time.

Both simulation and measurement results verify the effectiveness of the proposed method. The reconstructed array patterns maintain good agreement with the reference patterns under different beam-steering directions, demonstrating that data-driven adaptive grouping, all-on element-pattern acquisition, and sparse sampling can jointly reduce measurement effort while preserving reconstruction accuracy. Overall, the main contribution is a practical efficiency–accuracy tradeoff: the grouping is reduced automatically where fields vary slowly and refined where they vary rapidly, so fewer representative active element pattern measurements are needed without sacrificing the dominant beam features.

## Figures and Tables

**Table 1:** Comparison with related grouping strategies for the simulated 8×8 dipole antenna array.

Item	Representative Elements	Mean ESS (max)	Mean ESS (rms)	Mean 2D Pattern Similarity	AEP Acquisition Mode
3 × 3 grouping method in [14,15,16,17]	9/64	−25.96 dB	−36.39 dB	92.3909%	On-off
5 × 5 grouping method in [17]	25/64	−31.40 dB	−40.90 dB	94.7117%	On-off
Proposed method (10° sampling)	16/64	−25.53 dB	−38.85 dB	94.2419%	All-on
Proposed method	16/64	−42.01 dB	−49.76 dB	99.9995%	All-on

**Table 2:** Comparison with related grouping strategies for the measured 4 × 4 mmWave phased array.

Item	Representative Elements	Mean ESS (max)	Mean ESS (rms)	Mean 2D Pattern Similarity	AEP Acquisition Mode
3 × 3 grouping method in [14,15,16,17]	9/16	−18.91 dB	−26.01 dB	88.29%	On-off
Proposed method (10° sampling)	8/16	−20.96 dB	−27.90 dB	91.23%	All-on
Proposed method	8/16	−20.90 dB	−28.21 dB	92.13%	All-on

## Data Availability

The raw data supporting the conclusions of this article will be made available by the authors on request.
